# Neurocognitive Mechanisms of Impaired Decision Making in Pathological Gambling

**DOI:** 10.3389/fpsyt.2013.00012

**Published:** 2013-03-08

**Authors:** Simon Dymond, Natalia S. Lawrence, Kenneth S. L. Yuen

**Affiliations:** ^1^Department of Psychology, Swansea UniversitySwansea, UK; ^2^School of Psychology, University of ExeterExeter, UK; ^3^Neuroimaging Center, University Medical Centre MainzMainz, Germany

Recently, Power et al. (2012) used functional magnetic resonance imaging (fMRI) to investigate the neural systems underpinning the decision making performance of pathological gamblers (PG) and non-gamblers on the Iowa Gambling Task (IGT: Bechara et al., 1994). In the IGT, participants make selections from four decks of cards each associated with variable levels of monetary reward and loss. Two of the decks result in frequent immediate high gain, but produce high-magnitude losses of differing frequencies depending on the deck, leading to a cumulative long-term loss (“risky decks”). The remaining two decks typically result in lower immediate rewards, but also generate lower magnitude losses at the same frequency of punishment as the risky decks, resulting in a cumulative long-term gain (“safe decks”). Previous behavioral studies have found that gamblers usually show a preference for the risky decks (Goudriaan et al., 2005; Linnet et al., 2011).

According to Power et al. (2012), “the only fMRI study to date using a modified version of the IGT” (p. 625) was conducted by Tanabe et al. (2007). In that study, substance-dependent individuals were presented with computer-selected and participant-selected card trials, and greater activation in ventral medial frontal and right anterior prefrontal regions was found during active versus passive decision making in healthy controls only. However, substantial neuroimaging evidence, including fMRI, has clarified the distinct roles of prefrontal and other brain regions underlying the different aspects of successful, unimpaired IGT performance (Ernst et al., 2002; Fukui et al., 2005; Windmann et al., 2006; Christakou et al., 2009; Lawrence et al., 2009; Li et al., 2010; Bianchin and Angrilli, 2011; Gansler et al., 2012). For instance, Lawrence et al. (2009) used event-related fMRI and decomposed task components into decision making *per se*, risky versus safe choices, successful task learning, learning over time, and receipt of win versus loss outcomes. Findings showed that general decision making lead to activation in the ventromedial prefrontal cortex; wins more than losses evoked activation in striato-thalamic regions; choices from risky versus safe decks activated the medial frontal gyrus, lateral orbitofrontal cortex (OFC), and insula, and activation in these regions as well as pre-supplementary motor area (SMA) and secondary somatosensory cortex was positively correlated with IGT performance. Activation in lateral OFC and pre-SMA was modulated over time, indicating a general role in supporting task learning.

In their study, Power et al. (2012) hypothesized that the impaired learning of 13 gamblers on the IGT would be related to greater activation of OFC and striatum during risky choices. Findings revealed between-group differences in activation to risky versus safe choices in several clusters including right caudate nucleus, right frontal pole/OFC, amygdala, hippocampus, prefrontal cortex, and brainstem. There are, however, potential limitations to the procedures used and analyses undertaken which suggest a degree of caution should be exercised in interpreting these results.

The authors used an IGT where real task blocks alternated with baseline blocks where participants were instructed which cards to pick. This task may not have been optimal for use with fMRI. First, it is desirable to initially validate modified versions of standard tasks such as the IGT by collecting pilot behavioral data with healthy controls to determine whether or not the modifications influence learning (Lawrence et al., 2009). Second, control group performance is impaired by decision-phase time constraints, such as the 2 s time window for decisions used (Cella et al., 2007), which may explain why the overall score of the controls was lower than in other studies and not significantly different from the gamblers. Third, the brief interstimulus interval (0.5 s) and near-equal numbers of baseline and experimental trials per block may have lacked sufficient power to detect ongoing changes in BOLD. Although the authors compared activation to risky versus safe deck selections, such analyses confound deck type with outcome (i.e., the presentation or absence of reward and punishment), as well as learning stage (early or late). Fourth, the wins and losses presented were identical to the original IGT (Bechara et al., 1994) and, as a result, the regressor used (i.e., when participants chose from a deck) likely reflected not only a mixture of decision and outcome processing but also both win and loss processing.

Sub-optimal designs may impact on the possible delineation of different brain activation patterns in each group, which is further compounded by the fact that outcome was not examined separately. Lawrence et al. (2009) employed variable intertrial intervals consisting of a fixation cross that acted as a baseline for analysis, modified the reward and punishment schedules such that equal numbers of wins and losses were presented, and separated trials resulting in a win and a loss. These features permitted an analysis of the distinct role of prefrontal regions underpinning healthy IGT performance. Thus, although baseline trials were not included in Power et al.’s (2012) analysis, the absence of any reported pilot behavioral data comparing learning with and without the baseline task, combined with insufficient jittering to detect between-group changes in activation, means that the task may have confounded reward-based learning with instructed learning (Li et al., 2011). Reported findings showing activation in distinct prefrontal regions may thus not accurately reflect the unique demands exercised by the different task components (Gansler et al., 2012).

Finally, groups did not differ on any of the commonly employed IGT behavioral measures, yet confusingly the authors state, “control subjects did significantly differ from PG subjects in net winnings and from block to block” (p. 633). This statement is not supported by the data described in the results section. Moreover, significant differences were only revealed when data were converted to quartiles and a group · quartile interaction emerged. Furthermore, no correlations were reported between the behavioral measures and changes in BOLD response; indeed, behavioral performance was controlled for (as a “nuisance” covariate) in the fMRI analysis which makes interpretation of the functional significance of any differences in BOLD difficult.

Thus, while the findings of Power et al. (2012) are a noteworthy addition to the literature, the implications for understanding the neurocognitive mechanisms of impaired decision making in pathological gambling remain unclear.
